# Integrating GIS and local knowledge for optimised medical supply prepositioning in flood-prone Nigeria: a strategic framework

**DOI:** 10.1080/16549716.2026.2674432

**Published:** 2026-05-26

**Authors:** Maria Massironi, Modupe Jimoh, Jeremiah Ogah, Olatunji Matthew Kolawole, Martin Bounds

**Affiliations:** a Humanitarian Engineering, School of Engineering, University of Warwick, Coventry, UK; b Infectious Disease and Environmental Health Research Group, Department of Microbiology, Faculty of Life Sciences, University of Ilorin, Ilorin, Nigeria

**Keywords:** Flood preparedness, medical supply chains, Nigeria, emergencies, Hub Locator

## Abstract

**Background:**

Nigeria faces recurring flood disasters that severely disrupt its healthcare systems and delays medical relief efforts. Current medical supply prepositioning by the National Emergency Management Agency (NEMA) remains inadequate due to infrastructure limitations, funding constraints, and poor coordination.

**Objective:**

To develop and validate a Strategic Medical Hub Locator Framework that integrates Geographic Information Systems (GIS) analysis with qualitative local knowledge to optimise medical supply prepositioning for flood emergencies in Nigeria.

**Methods:**

We employed a mixed-methods approach, combining qualitative analysis of 22 GIS-based flood risk studies in Nigeria and structured expert survey responses from 34 professionals with flood response experience, including five with direct NEMA warehouse experience. The framework was grounded in Disaster Risk Reduction theory and supported by Protection Motivation Theory and Theory of Planned Behaviour.

**Results:**

The framework identified eight strategic medical hub locations: six existing NEMA warehouses (Abuja, Lagos, Kano, Gombe, Maiduguri, Kaduna) with the strongest performance, and two new strategic sites (Akure and Uyo) to address service gaps in South–South and South–East regions. Abuja emerged as the highest-performing hub with a composite score reflecting good centrality, governance capacity, and operational readiness. The framework successfully bridged data silos between spatial flood risk patterns and operational constraints.

**Conclusions:**

Integration of GIS thematic analysis with qualitative local data provides a viable, replicable method for enhancing humanitarian medical logistics in flood-prone regions. The Strategic Medical Hub Locator Framework offers a structured decision-support tool that can inform evidence-based investment in Nigeria’s disaster health infrastructure, while being adaptable to other Global South contexts.

## Paper Context

- **Key findings**: Where primary geospatial data silos are present, GIS analysis can be enhanced by combining it with local qualitative insights, thereby bridging the gap between technical modelling and practitioner realities.
- **Added knowledge**: The paper offers a methodological contribution through the design of a Strategic Medical Hub Locator Framework that is replicable in contexts where primary data and complex modelling are not feasible.
- **Global health impact for policy and action**: This study provides policymakers and humanitarian actors with a proof-of-concept decision-support tool. The exploratory framework offers a scalable model that can be adapted to other flood-prone settings where primary data and practical knowledge are scattered, thereby strengthening disaster preparedness and health system resilience strategies.

## Background

Climate change has progressively intensified extreme weather events, with flooding now recognised as one of the most severe recurring disasters globally, consistently causing significant loss of life and displacement [1,2]. Nigeria is especially vulnerable, experiencing disproportionate flood impacts in comparison with other African countries. Between 2011 and 2020, Nigeria accounted for approximately 15% of all flood-related African deaths and 21% of continental property damage [1]. The devastating 2012 flood event alone displaced 2.1 million people and caused $16.9 billion USD in economic losses [3,4].

Despite substantial humanitarian efforts and national disaster response frameworks, Nigeria’s medical response to flood crises remains systematically delayed [4,5]. The National Emergency Management Agency (NEMA) has established 16 warehouses across 14 locations for emergency supply prepositioning, yet consumable medical supplies remain inadequately prepositioned due to weak infrastructure, funding gaps, and poor coordination [6,7]. Current warehousing systems lack sufficient cold-chain capacity, medical inventory tracking, and strategic geographic optimisation for flood-specific scenarios.

Global research demonstrates that data-informed prepositioning strategies can significantly reduce emergency response times and improve aid efficiency [8]. However, Geographic Information Systems (GIS) approaches, while valuable for mapping flood risks and optimising facility placement, often lack the qualitative local information needed to reflect operational realities [9,10]. In Nigeria, this disconnect between technical spatial analysis and ground-level constraints, combined with data silos, has contributed to suboptimal supply placement, stock wastage, and delivery delays [11,12].

The objective of this study is to address these critical gaps by developing an exploratory Strategic Medical Hub Locator Framework that systematically integrates GIS evidence with qualitative local insights. Location efficiency was identified based on perceived operational accessibility, responsiveness, timeliness, and lack of underutilisation. Our approach differs from previous spatial optimisation models by explicitly incorporating stakeholder perceptions, operational constraints, and institutional knowledge into the decision-making process. We hypothesise that integrating GIS technology with qualitative local data can significantly enhance the strategic prepositioning of medical supplies in Nigeria’s flood-prone areas.

## Methods

### Study design

**Figure 1.**
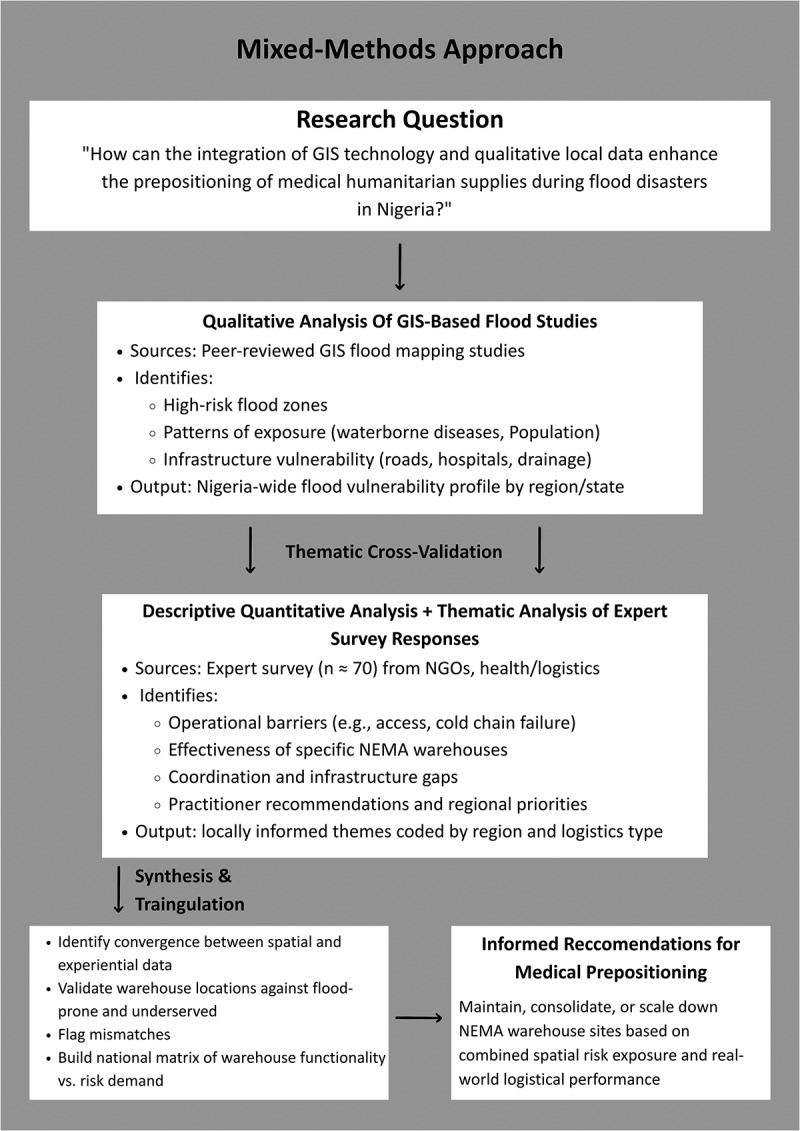
Mixed-methods interaction model for enhancing medical supply prepositioning in flood-prone Nigeria.

We adopted a mixed-methods research design, combining qualitative analysis of GIS-based flood studies with a survey analysed using descriptive quantitative analysis and thematic analysis, allowing triangulation across data sources [13,14] (Figure 1). The methodology comprised (1) systematic qualitative analysis of existing GIS-based flood studies in Nigeria and (2) structured expert survey incorporating structured expert judgement (SEJ) methodology principles [13,14]. The study followed the STROBE reporting guideline for cross-sectional research. A completed STROBE checklist is provided as a supplementary file. The GIS-based flood studies were qualitatively analysed to identify where floods have the most *documented* logistical and health impacts despite flood frequency. Concurrently, the expert survey data were analysed using descriptive quantitative methods to summarise response distributions, while thematic analysis was applied to identify recurring topics and insights. Survey findings were reported with parenthetical indications of response frequency to descriptively convey the relative prominence of themes. Interpretation is, therefore, qualitative in nature and grounded in expert judgement.

### GIS literature analysis

We systematically reviewed GIS-based flood risk studies published from 2000 onwards that examined flood risks and predictability across Nigeria. Selection criteria included (1) national or multi-state scope, (2) explicit reference to flooding, (3) GIS-based or hybrid methodology, and (4) peer-reviewed publication. Twenty-two studies met the inclusion criteria, covering 374 discrete flood risk references across Nigerian states.

Studies were coded using NVivo for (1) geographic focus by state and zone, (2) flood drivers and severity, and (3) relevance to logistics and health outcomes. This approach enabled mapping of spatial flood risk patterns while identifying areas where floods most severely disrupt healthcare access and essential services.

### Expert survey

A structured online Qualtrics survey was distributed from June to August 2025 to professionals with field experience in flood response, disaster logistics, or emergency medical planning in Nigeria. Inclusion criteria required: (1) minimum 2 years’ experience in flood/disaster response, humanitarian logistics, or public health planning in Nigeria, (2) organisational affiliation with relevant agencies, and (3) direct experience in Nigerian flood response or disaster management positions.

The 29-question expert survey addressed five thematic areas: feasibility, effectiveness, coverage, optimisation, and prioritisation of the current warehouse networks. Closed-ended questions were analysed quantitatively using frequencies, rankings, and composite scoring. Open-ended responses were analysed using thematic analysis to identify recurring patterns on topics like access gaps, delay mentions, and underserved zones. A target of approximately 70 expert participants was set to ensure thematic saturation and statistical significance for subgroup comparisons across Nigeria’s six geopolitical zones [14]. A total of 59 responses were received. The incomplete survey responses were excluded listwise; no imputation was applied. Responses that did not meet the inclusion criteria were also excluded. Accordingly, 34 responses were complete and analysable, including 5 respondents with direct NEMA warehouse experience. This number was deemed adequate for exploratory analysis and qualitative triangulation. The full expert survey, NVivo coding framework, and thematic operationalisation scheme are provided in Appendix.

### Mixed-methods integration

Findings from the GIS-based flood risk qualitative synthesis and the structured expert survey were integrated through a triangulation process. Convergent evidence from both datasets (for example, North–Central and South–South zones being identified as recurrent high-risk and underserved regions) was prioritised in the framework. Divergent insights, like discrepancies between GIS-identified high-risk areas and expert prioritisation of certain hubs, were examined to contextualise variations in local experience and risk perception. This integration allowed for the cross-verification of insights to reduce potential bias arising from data silos, thereby ensuring that spatial modelling data were grounded in practitioner realities. The combined insights directly informed the Strategic Medical Hub Locator Framework’s composite scoring system and the identification of eight strategic medical hub locations presented in the Results section.

### Framework development

**Figure 2.**
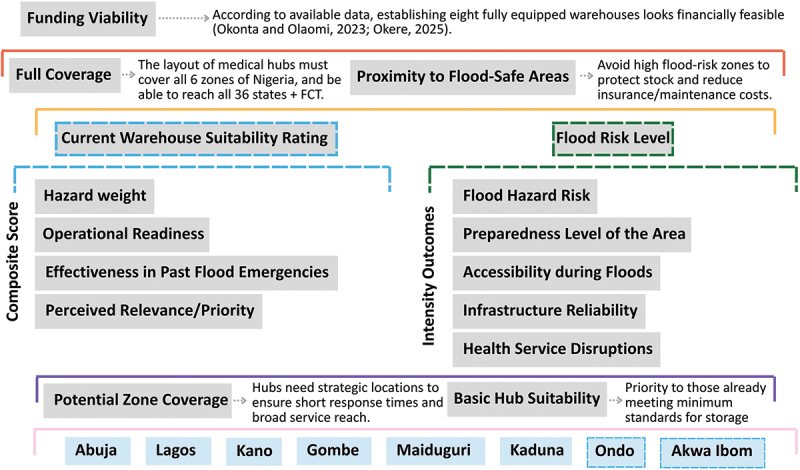
Strategic medical Hub Locator Framework [17,18].

The Strategic Medical Hub Locator Framework synthesised findings through four integrated levels: (1) funding viability, (2) coverage and safety considerations, (3) current warehouse suitability and flood risk assessment, and (4) zone coverage and suitability (Figure 2, Table 1). A composite scoring system was developed to provide a structured way to translate the qualitative data collected into a reproducible metric by pulling heterogeneous evidence into a single, comparable value:

**Table 1.** Strategic medical Locator framework legend.

Funding viability	Availability and sustainability of financial resources to establish and maintain a warehouse/warehousing network.
Full covergae	The warehouse/warehousing network’s capacity to provide adequate geographic and population coverage over the entire territory (not only flood-prone areas).
Proximity to flood safe-areas	The closeness of a warehouse site to elevated or flood-resilient zones that remain accessible during flood events to reduce the risk of operational disruption.
Current warehouse suitability rating	The weighting of the variables: hazard weight, operational readiness, effectiveness in past flood emergnecies, perceived relevance/priority.
Hazard weight	This value represents the intensity of the physical threat that a warehouse is expected to face/mitigate.
Operational readiness	The ability of the site to become functional rapidly during emergencies, considering logistics systems, equipment availability, and emergency protocols.
Effectiveness in past flood emergnecies	The historical performance of the location or facility in previous flood responses, including timeliness, reliability, and impact of medical supply distribution.
Perceived relevance/priority	Stakeholder and expert judgement regarding the strategic importance of the location within the flood response and logistics network.
Flood risk level	The weighting of the variables: flood hazard risk, preparedness level of the area, accessibility during floods, infrastructure reliability, health service disruptions.
Flood hazard risk	The flood characteristics like depth, duration, and frequency of a specific area
Preparedness level of the area	The general readiness of an area to respond to floods like local disaster planning and emergency response based on historic data.
Accessibility during floods	The reliability of transport routes and physical access to the site during flood events.
Infrastructure reliability	Evaluates the robustness of critical infrastructure like power supply, water, and communications.
Health service disruptions	The extent to which floods are likely to interrupt nearby health services.
Potential zone	Warehouse locations that fall within zones enabling broad service coverage and short emergency response times during flood events.
Basic hub suitability	Prioritisation for potential upgrades of warehouses that already meet minimum threshold criteria across multiple logistical, infrastructural, and hazard-related factors.

Composite score = Hazard weight + Operational readiness + Effectiveness +2 × Rank-#1 + Priority-zone count – Underserved-zone – Lack-access state – Delay mentions

Each component reflects a different aspect of warehouse performance and strategic value based on survey responses and GIS analysis findings. The factors within the composite score were quantified by counting the number of expert survey responses that cited them, so the numerical values directly reflect how often participants identified that criterion as relevant (Table 2). The weighting structure of the composite scoring approach was designed to prioritise transparency and minimise arbitrary assumptions. The relative importance of each factor was inferred from expert responses by using their selections as a proxy for operational relevance. Variables were treated with equal importance due to the exploratory nature of the framework and the absence of established weighting schemes for this context. The ‘Rank-#1’ variable was given double weight (×2) to ensure that dominant option preferences exert greater influence on the final ranking [15]. This is consistent with extensions of the TOPSIS method that prioritise ranking stability and dominant preferences under conditions of uncertainty or incomplete information [15]. Since 34 complete responses were collected, each factor could range from zero (no respondent mentioned it) to 34 (all respondents cited it), except for the ‘Rank-#1’ variable as its maximum possible contribution was 68.

**Table 2.** Strategic Medical Hub composite scores and performance metrics.

Location	Warehouse	*F*_severity	Op Ready	Effectiveness	Rank	Underserved	No Access	Priority	Composite
FCT Abuja	Abuja	Moderate	6	2	1	8	0	8	High
Lagos	Lagos	Moderate	2	2	1	0	2	0	High
Borno	Maiduguri	Negligible	1	2	2	3	0	3	High
Kaduna	Kaduna	Moderate	0	1	0	1	0	1	Moderate
Kano	Kano	Very high	0	0	0	1	1	1	Moderate
Gombe	Gombe	Moderate	0	0	0	3	0	3	Moderate
Ondo		Low	0	0	0	0	0	0	Low
Akwa Ibom		Low	0	0	0	8	1	7	Negligible

### Data analysis

Both analysis streams were coded using NVivo and analysed using Excel. GIS literature findings were systematically compared with survey responses to identify convergent themes and divergent priorities. Spatial and thematic insights were evaluated against known NEMA warehouse locations to formulate evidence-based recommendations for medical prepositioning optimisation.

### Ethical considerations

This study received full ethical approval from the Delegated Engineering Ethics Committee (DEEC), a subcommittee of the Biomedical and Scientific Research Ethics Committee (BSREC), University of Warwick (Application Reference: DEEC019/24-25). All survey participants provided digitally written informed consent, and responses were anonymised to protect participant confidentiality. The research was conducted in compliance with the University of Warwick’s Research Code of Practice, relevant data protection legislation, and in accordance with the principles of the Declaration of Helsinki.

## Results

The qualitative analysis revealed that River Overflow/Dam Release and Heavy Rainfall constitute the dominant flood drivers across Nigeria, with particular concentration in the Niger-Benue and Sokoto-Rima basins. Based on the frequency and richness of references, the analysis of 374 flood risk references revealed a clear pattern of thematic concentration, expressed as South–West > South–South > North–Central > North–West > North–East > South–East. At the state level, Kogi, Anambra, and Bayelsa emerged as recurrent high-risk areas, with Kogi being especially prominent due to its strategic position at the Niger-Benue confluence and role as Nigeria’s communication network hub [16]. Transportation/Infrastructure Impact represented the greatest intensity of concern regarding functional loss and aid delays, particularly affecting Kano, Jigawa, and Rivers states.

**Figure 3.**
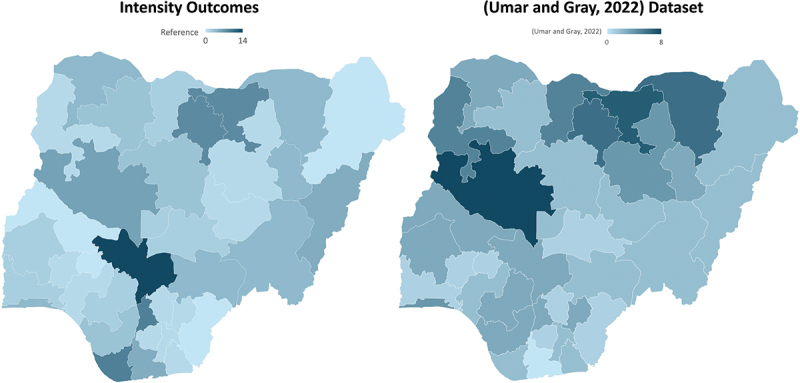
Comparison of GIS analysis findings with Umar and Gray (2022) flood event dataset [1]. It highlighted the alignment between states with high flood frequencies [1] and impact relevance as identified by the flood documentation of logistical and health impacts.

Comparison with external datasets, including Umar and Gray’s (2022) recorded flood events, demonstrated alignment between GIS-derived risk patterns and empirically documented flood impacts [1] (Figure 3). Both analyses identified the vulnerability of major river basin states, such as Niger, Jigawa, and Kano, reinforcing the validity of the GIS-based qualitative synthesis. Differences in ranking, most notably Kogi’s stronger emphasis in this study despite fewer recorded events in Umar and Gray’s (2022), reflected the inclusion of contextual factors like its strategic position at the Niger-Benue confluence and its role as a national transport hub [16]. These comparisons confirmed the framework’s capacity to successfully identify areas where high physical flood exposure translates into operational disruption, validating the integration approach.

Among 34 analysable responses, only five respondents reported direct NEMA warehouse experience, highlighting limited operational knowledge across the relevant broader humanitarian community. Key infrastructure limitations identified included security concerns (reported by seven respondents), poor road access during rainy seasons (six respondents), and lack of trained logistics staff (six respondents). Regarding warehouse effectiveness, proximity to high-risk flood zones (13 responses) and good local coordination (12 responses) were prioritised over technical factors, such as cold storage capacity (five responses). Twenty-two respondents considered the current 16-warehouse network insufficient, with preferences for hybrid models combining well-equipped strategic hubs with regional basic depots. Geographic analysis revealed significant coverage gaps, with North-Central (eight responses) and South–South (eight responses) zones identified as most underserved. Specific states lacking timely access included Kogi (four mentions), Niger State (four mentions), and Kwara (two mentions). The full framework assessment of flood risk, operational capacity, and coverage needs identified a network of eight strategic medical hub locations. These comprise high- and moderate-performing existing warehouses identified through composite scoring, as well as additional new locations identified through the broader framework assessment (Table 2).

High-performing existing hubs:

Based on the composite scoring, the following existing facilities emerged as high-performing hubs:

- **Abuja (FCT)**: Achieved the highest composite score reflecting optimal centrality, governance capacity, and institutional coordination.
- **Lagos**: Strong operational readiness and effectiveness despite moderate flood exposure.
- **Maiduguri**: High strategic value for North-East coverage with acceptable risk profile.

Moderate-performing existing hubs:

These facilities reflected adequate baseline capacity with some identifiable operational/hazard-related constraints:

- **Kaduna, Gombe**: Demonstrated adequate capacity but with specific operational constraints.
- **Kano**: Despite operational constraints, demonstrated high priority and strategic localisation.

Low or negligible composite-score potential new hubs:

These locations recorded low or negligible composite scores because several variables contributing to the composite assessment are specific to existing warehouse infrastructure and performance. As reflected in the framework design, composite scoring prioritises the strengthening of existing, well-functioning facilities and networks rather than the creation of entirely new ones. This is because upgrading established infrastructure ensures lower implementation costs and reduced coordination risk compared to establishing new facilities from scratch. Consequently, the low composite scores do not indicate low strategic relevance, as these locations demonstrate considerable strategic value when assessed across the full set of framework variables, including flood risk patterns, service coverage potential, accessibility, and expert judgement.

- **Akure (Ondo State)**: Selected to address South–West service gaps with low flood severity risk.
- **Uyo (Akwa Ibom State)**: Strategically positioned to improve South–South and South–East coverage.

**Figure 4.**
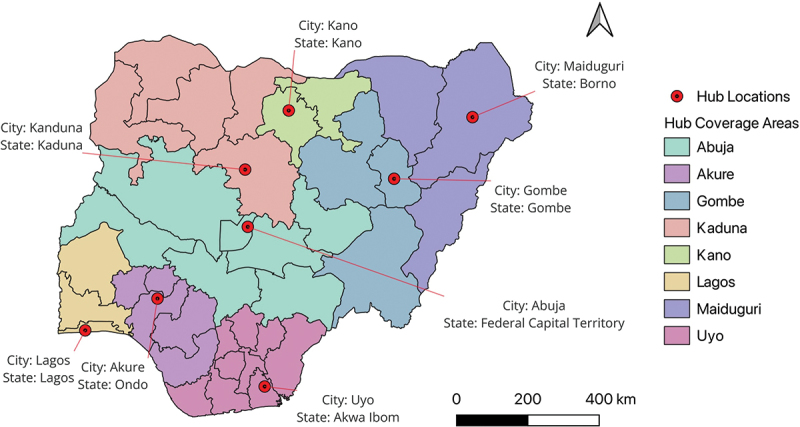
Map showing distribution of recommended medical hubs across Nigeria.

The eight-hub network provides comprehensive national coverage while maximising operational efficiency and minimising environmental risks (Figure 4).

## Discussion

This study demonstrates that systematic integration of GIS thematic analysis with local qualitative knowledge can bridge critical gaps in humanitarian medical logistics planning. The Strategic Medical Hub Locator Framework successfully synthesised flood hazard mapping with operational constraints to produce actionable recommendations for medical supply prepositioning in Nigeria.

The identification of Abuja as the highest-performing hub underscores the importance of centrality and governance capacity in effective prepositioning, consistent with literature emphasising accessibility and institutional coordination for rapid response [5,8]. Conversely, the framework’s identification of Akure and Uyo highlights its capacity to address regional inequities by integrating hazard exposure with logistical accessibility considerations.

Previous studies in flood risk management and humanitarian logistics predominantly relied on either technically driven GIS-based approaches or expert- and participation-led methods in isolation. Purely technical models tend to prioritise hazard frequency, spatial exposure, or optimisation metrics derived from modelled and compiled datasets but may inadequately capture institutional capacity, coordination issues, and other accessibility constraints in humanitarian settings [1,10]. Conversely, expert- and participatory approaches emphasise contextual and operational knowledge but face challenges in systematic spatial integration and standardisation when not combined with analytical modelling [9,11]. These distinctions align with two dominant themes in humanitarian facility location modelling: data-driven optimisation models and qualitative or expert-informed decision frameworks. In contrast, the proposed framework explicitly integrates both approaches. It combines GIS-based spatial analysis with practitioner-derived insights to support decision-making under data and/or operational constraints. The composite scoring methodology enables transparent integration of multiple evidence streams while maintaining adaptability across contexts and varying data availability.

Our findings suggest that concentrating resources on fewer, strategically optimised locations may be more effective than simply maintaining the current dispersed network of 16 warehouses. Available data seem to hint at a shift towards heavier investment in NEMA flood preparedness [17]. This includes the National Economic Council’s ₦10 billion NGN allocation to NEMA for 2025 [17]. Additionally, a recent study shows that, in Nigeria, a fully equipped flood-relief logistics network can operate effectively at around $1 million USD per season [18]. Based on these data and survey responses, the framework’s recommendation of eight strategically distributed cold-chain warehouses supported by existing ambient ones appears financially feasible.

The identification of specific coverage gaps in North–Central and South–South regions provides actionable intelligence for immediate policy interventions. The framework’s emphasis on security, coordination, and accessibility over purely technical specifications aligns with expert priorities and operational realities. Overall, the framework is exploratory and not intended as a definitive policy prescription. Nonetheless, it provides a structured decision-support tool that can inform national disaster preparedness planning in Nigeria. Specifically, it can support the identification of priority locations for investment, guide the rationalisation of the existing warehouse network, and highlight geographic coverage gaps that require targeted intervention.

While developed for Nigeria, the Strategic Medical Hub Locator Framework addresses broader challenges facing flood-prone regions globally. The methodology’s emphasis on integrating technical spatial analysis with local knowledge reflects growing recognition of participatory approaches in disaster risk reduction [9,11]. By integrating spatial risk patterns with operational insights, the framework may assist policymakers in aligning infrastructure upgrades, coordination mechanisms, and resource allocation with evolving flood risk and population needs. The framework’s modular design enables adaptation to different institutional contexts, data availability scenarios, and risk profiles.

### Limitations and delimitations

Several limitations warrant consideration. First, the expert survey achieved lower participation than targeted, with only five respondents having direct NEMA warehouse experience. This limited sample size, while providing valuable insights, reduces the statistical generalisability of findings. Second, the framework relied on secondary GIS analysis rather than original spatial modeling, limiting technical precision. With enhanced data availability, the framework could be validated through primary spatial modelling or quantitative analyses to assess ranking robustness. Third, detailed operational data on infrastructure capacity, staffing levels, and transport accessibility were unavailable, constraining the framework’s specificity.

The framework should therefore be considered exploratory rather than definitive. Its focus was on demonstrating feasibility and approach rather than providing a fully validated national model. Future applications would benefit from expanded expert participation, primary spatial data collection, and incorporation of additional operational variables.

### Ethical/operational reflections

Our integration approach aimed to balance technical information with lived realities, ensuring that findings remain both evidence-based and contextually grounded. The study’s limited sample of experts raises an ethical responsibility to represent practitioner perspectives fairly. The incomplete operational data necessitated caution regarding existing data gaps to inform recommendations. To mitigate uncertainty about results, we cross-checked survey insights against peer-reviewed literature and documentation to validate patterns where possible.

### Future research directions

Several avenues merit further investigation. First, longitudinal validation studies could assess framework performance over multiple flood seasons. Second, comparative applications in other flood-prone countries could evaluate transferability and cultural adaptability. Similarly, the framework structure’s adaptability to other climate and health emergencies could be explored through context-specific criterion adjustments. Third, integration with climate change projections and economic evaluation could enhance long-term strategic planning capacity. Finally, responses from affected communities could develop the framework as a localised adaptive strategy.

## Conclusions

This study demonstrates that integrating GIS technology with qualitative local knowledge provides a viable and replicable approach for enhancing humanitarian medical logistics in flood-prone regions. The Strategic Medical Hub Locator Framework successfully bridged gaps between technical spatial analysis and operational realities, producing evidence-based recommendations for optimising Nigeria’s medical supply prepositioning infrastructure.

For Nigeria, the Framework highlights eight priority hubs, offering actionable guidance for NEMA. The methodology’s emphasis on stakeholder engagement and operational constraints provides a model for inclusive, context-sensitive disaster risk reduction planning. While exploratory in scope, the Framework offers one pathway towards more evidence-based, efficient, and equitable disaster health preparedness. Ultimately, this proof-of-concept demonstrates significant potential for adaptation in other Global South contexts where data and operational knowledge remain fragmented.

## Supplementary Material

STROBE.doc

Supplementary file.docx

## Data Availability

The primary data used for this study cannot be shared publicly due to ethical restrictions specified in the approved consent form. Relevant supporting data necessary to reproduce or confirm the validity of the findings are included in the Appendix. For any additional concerns or data requirements, the authors can be contacted directly.
