# Supplementary material for: Integrating GIS and local knowledge for optimised medical supply prepositioning in flood-prone Nigeria: a strategic framework
Source: Glob Health Action. 2026 May 26;19(1):2674432. doi: 10.1080/16549716.2026.2674432 (PMC13215400; doi:10.1080/16549716.2026.2674432)
Supplement: Supplementary file.docx [file ZGHA_A_2674432_SM3863.docx]

**Appendix**

## *Table A: Data Selection Criteria of GIS-based Flood Risk Qualitative Study (Source: Authors own work)*

| **Factors** | **GIS-based Flood Risk Qualitative Study Criteria** |
| --- | --- |
| Location | - Focus on Nigeria - National-scale or multi-state studies |
| Timeline | - Use data from the 2000s onward - Use data that extends beyond the early 2000s - Ideally include around 20 years of data (e.g., 2000–2020 or longer) |
| Topic | - Must explicitly refer to flooding or flood risk/predictability - May include related topics like waterborne diseases if clearly connected to flood conditions |
| Methodology | • Must be GIS-based or use a GIS hybrid approach |
| Source Type | - Must be a scholarly article - Available in academic repositories or databases |
| Language | • English |
| Accessibility | • Full text should be accessible through open access or institutional access |
| Quality Preference | • Studies with clearly stated methods, heavy GIS use, and regional disaggregation are preferred |

## *Table B Coding Matrix of GIS-based Flood Risk Qualitative Study (Source: Authors own work)*

| Name | Description | Sample | Source | Reference |
| --- | --- | --- | --- | --- |
| Flood Drivers and Severity | Parent Code: Anything related to flood causes | “The rainy season typically spans from March to October, bringing substantial rainfall that affects the region's hydrological dynamics. This rainfall is crucial for replenishing water resources but can also exacerbate flooding, particularly in lower elevation zones…Urbanization in the Udi, Ezeagu, and Ojir River LGAs is growing, with increased development in towns and rural areas. This urban expansion affects land use patterns, water management, and environmental sustainability. The interaction between urbanization and natural processes requires careful management to mitigate impacts such as increased runoff, erosion, and habitat disruption.” [20] | 22 | 374 |
| Building on Natural Waterways | Refers to building infrastructure atop or within naturally occurring water channels like rivers, creeks, or streams [19] | “In developing nations such as Nigeria, natural elements like excessive rainfall and soil characteristics are the major causes of ﬂooding (Malcolm, 2015), however, the ﬂood impacts are more severe due to rapid urbanization, poor infrastructure, and inadequate environmental planning” [21] | 19 | 50 |
| Climate Change | Climate change refers to long-term shifts in temperatures and weather patterns, primarily caused by human activities, especially the burning of fossil fuels like coal, oil, and gas [23] | “The frequency and severity of climate-related disasters,  including heat waves, droughts, storms, and ﬂoods, have increased, and there is a strong connection between these events and human-caused greenhouse gas emissions.” [22] | 16 | 29 |
| Deforestation and Land Degradation | Deforestation is the permanent removal of forest cover for other land uses, while land degradation is the decline in land quality and productivity caused by natural or human activities [24]. | “The main cause of flooding according to [7], is excessive rainfall, but however, there are many other causes resulting from human activities, for example: land degradation, deforestation of catchment areas…” [25] | 13 | 25 |
| Heavy Rainfall | Heavy rainfall refers to precipitation with a high intensity which often leads to flooding or reduced visibility [27]. | “Higher rainfall in the southern part of the study area and varied elevation also influence flood risk distribution. High-risk regions need robust flood defenses and efficient drainage systems. Sustainable land management practices and advanced flood control measures are essential for mitigating flood risk, particularly in high rainfall and steep slope areas.” [28] | 20 | 53 |
| Poor Infrastructure | Poor infrastructure refers to inadequate or substandard physical systems and facilities essential for a society or economy, such as roads, power, water, sanitation, and communication networks, which hinder development, reduce efficiency, and limit access to basic services [26] | “Establishing sustainable flood management has been a public policy priority due to the widespread lack of essential services and infrastructure in many communities, making them vulnerable to these natural disasters” [29] | 16 | 43 |
| River Overflow - Dam Release | Flooding that happens when intense or prolonged rainfall rapidly raises river levels, or when water is suddenly discharged from an upstream reservoir causing the riverbed/channel to no longer contain the flow and to overtop its banks [30] | “In recent years, the Niger River basin in Nigeria has experienced increasing ﬂooding, particularly affecting the northern regions such as Kogi and Nasarawa States. The ﬂood disasters of 2012 and 2022 are regarded as the worst in recent times.” [22] | 21 | 59 |
| Geographic Focus - Nigeria | Parent Code: Nigerian location where heavy flooding is mentioned. | “Abstract Floods are water induced disasters that lead to temporary inundation of dry land and cause serious damages in the affected location such as loss of lives and properties and destruction of infrastructures. They have become common occurrences in every part Nigeria and the recorded impacts of flooding on the inhabitants are alarming, causing hundreds of deaths and rendering thousands homeless.” [31] | 22 | 374 |
| North-Central | Heavy flooding is mentioned in relation to North-Central locations and states that belong to the zone: Federal Capital Territory (Abuja), Benue State, Kogi State, Kwara State, Nasarawa State, Niger State, Plateau State [32] | “Results indicate over 50% rise in inundation, with 15,000 hectares of vegetation and 143,000 residents enduring impacts. Attributing factors include elevated antecedent rainfall versus historical medians coupled with accelerating catchment modifications expanding runoff.” [33] | 14 | 33 |
| North-East | Heavy flooding is mentioned in relation to North-Eastern locations and states that belong to the zone: Adamawa, Bauchi, Borno, Gombe, Taraba, Yobe [32] | “The area is characterized by erosional and flood plains with several stream channels, and it is situated in the upper Benue trough catchment of the Yola arm. Seasonal flooding causes these erosional plains and floods, which are naturally rich in organic nutrients” [33] | 9 | 22 |
| North-West | Heavy flooding is mentioned in relation to North-Western locations and states that belong to the zone: Jigawa, Kaduna, Kano, Katsina, Kebbi, Sokoto, Zamfara [32] | “Sokoto River (2006), and Ogun River (2007), have exceeded their banks, drowning nearby houses on their floodplains.” [34] | 11 | 24 |
| South-East | Heavy flooding is mentioned in relation to South-Eastern locations and states that belong to the zone: Bia, Anambra, Ebonyi, Enugu, Imo [32] | “Low elevation zones are prone to flooding due to flat terrain and slow water movement, whereas higher elevations in Udi Hills show rapid runoff, increasing erosion risks.” [36] | 10 | 19 |
| South-South | Heavy flooding is mentioned in relation to South-Southern locations and states that belong to the zone: Akwa, Ibom, Bayelsa, Cross River, Delta Edo River [32] | “High-risk areas cover a substantial 5905.62 km2, indicating the regions most likely to experience significant flooding. Medium-risk areas, which face less-severe but still noteworthy flooding, span 8304.57 km2 of Delta State.” [37] | 16 | 34 |
| South-West | Heavy flooding is mentioned in relation to South-Western locations and states that belong to the zone: Ekiti, Lagos, Ogun, Ondo, Osun, Oyo [32]. | “The topography of Lagos (Figure 5) indicated the pattern of the city terrain to be relatively flat in the centre and it also has few meters high above sea level especially in the south-eastern areas; 3) of the 20 local government areas Lagos state (Figure 6) only two (Agege and Ifako) were found not to be at risk of flooding.” [38] | 15 | 49 |
| Relevance to Logistics and Health Outcomes | Parent Code: Any mention of relief logistics, flood management and prevention, warehousing and medical outcomes. | “Findings indicate that intensified rainfall will exacerbate erosion, soil degradation, and agricultural losses, threatening local livelihoods. Effective flood management strategies, including improved drainage, afforestation, and soil conservation, are crucial for mitigating these impacts. The study underscores the importance of climate adaptation policies to enhance agricultural resilience and sustainability in the region. Future research should refine predictive models and explore community-based adaptation measures for long-term flood mitigation.” [39] | 22 | 374 |
| Access to Essentials | The ability of affected populations to obtain life-sustaining goods and services within an acceptable timeframe after a disaster [40]. | “Abstract Floods are water induced disasters that lead to temporary inundation of dry land and cause serious damages in the affected location such as loss of lives and properties and destruction of infrastructures. They have become common occurrences in every part Nigeria and the recorded impacts of flooding on the inhabitants are alarming, causing hundreds of deaths and rendering thousands homeless.” [31] | 18 | 34 |
| Emergency Relief Distribution | First-wave logistics activities that move relief items close to the affected population, usually under time, information and infrastructure constraints [26] | “This flood level mapping is intended to enhance awareness among residents, prioritize land development, and improve emergency preparedness, including aid and relief operations in areas at high risk of flooding in the future.” [34] | 15 | 24 |
| Recovery Planning | The set of strategic and operational actions that guide the restoration and improvement of social, economic and health systems and infrastructures after the immediate response phase [42]. | “We can establish that machine learning techniques can effectively map and predict ﬂood-prone areas and serve as a tool for developing ﬂood mitigation policies and plans.” [41] | 18 | 52 |
| Transportation or Infrastructure Impact | The extent to which floods damage or disrupt physical infrastructure and the dripple-down effects on mobility, supply chains and health-care access [43]. | “Roads and bridges, particularly those adjacent to the shore and flood zones, are at danger of inundation during major flooding events, which has substantial consequences for the status of key infrastructure.” [35] | 15 | 32 |
| Warehousing | The strategic pre-positioning, storage, inventory control and outbound staging of relief supplies so they can be dispatched rapidly when a disaster strikes [45].  ==Due to lack of data, modified to==  Any information potentially relevant to instructing warehouse positioning | “This method ensures a smooth and secure evacuation process, minimizing congestion and potential risks to evacuees (Karna et al., 2023).” [44] | 8 | 9 |

## *Table C: Results of Flood Risk Qualitative Study: Flood Drivers by Zone (Source: Authors own work)*

|  | Build on Natural Waterways | Climate Change | Deforestation and Land Degradation | Heavy  Rainfall | Poor Infrastructure | River Overflow/Dam Release |
| --- | --- | --- | --- | --- | --- | --- |
| North-Central | 6 | 3 | 2 | 10 | 3 | 7 |
| North-East | 5 | 2 | 2 | 9 | 2 | 6 |
| North-West | 5 | 1 | 2 | 11 | 4 | 9 |
| South-East | 2 | 0 | 1 | 3 | 3 | 9 |
| South-South | 7 | 3 | 3 | 5 | 5 | 6 |
| South-West | 4 | 1 | 1 | 4 | 5 | 10 |

## *Table/Figure D1, D2: Results of Flood Risk Qualitative Study: Flood Drivers by State (Source: Authors own work)*

|  | Building on Natural  Waterways | Climate Change | Deforestation and Land Degradation | Heavy  Rainfall | Poor Infrastructure | River Overflow - Dam Release |
| --- | --- | --- | --- | --- | --- | --- |
| Benue | 0 | 0 | 0 | 1 | 0 | 1 |
| FCT Abuja | 0 | 1 | 0 | 1 | 0 | 1 |
| Kogi | 1 | 1 | 0 | 2 | 1 | 1 |
| Kwara | 0 | 0 | 0 | 0 | 0 | 0 |
| Nasarawa | 0 | 0 | 0 | 1 | 0 | 1 |
| Niger State | 0 | 0 | 0 | 1 | 1 | 1 |
| Plateau | 0 | 0 | 0 | 1 | 0 | 1 |
| Adamawa | 0 | 0 | 0 | 0 | 0 | 0 |
| Bauchi | 0 | 0 | 0 | 0 | 0 | 0 |
| Borno | 0 | 0 | 0 | 0 | 0 | 0 |
| Gombe | 2 | 0 | 0 | 0 | 0 | 0 |
| Taraba | 0 | 0 | 0 | 0 | 0 | 0 |
| Yobe | 0 | 0 | 0 | 1 | 0 | 0 |
| Jigawa | 0 | 0 | 0 | 2 | 1 | 2 |
| Kaduna | 0 | 0 | 0 | 2 | 1 | 1 |
| Kano | 1 | 1 | 1 | 1 | 0 | 1 |
| Katsina | 0 | 1 | 1 | 2 | 1 | 2 |
| Kebbi | 0 | 0 | 0 | 1 | 0 | 1 |
| Sokoto | 0 | 1 | 0 | 2 | 1 | 1 |
| Zamfara | 0 | 1 | 0 | 2 | 0 | 2 |
| Abia | 0 | 0 | 0 | 1 | 0 | 1 |
| Anambra | 0 | 0 | 0 | 1 | 0 | 2 |
| Ebonyi | 0 | 0 | 0 | 1 | 0 | 1 |
| Enugu | 0 | 0 | 0 | 0 | 0 | 0 |
| Imo | 0 | 0 | 0 | 0 | 0 | 1 |
| Akwa ibom | 0 | 0 | 0 | 1 | 0 | 1 |
| Bayelsa | 1 | 1 | 1 | 1 | 2 | 3 |
| Cross River | 0 | 0 | 0 | 0 | 0 | 0 |
| Delta | 0 | 0 | 0 | 1 | 0 | 1 |
| Edo | 0 | 0 | 0 | 1 | 0 | 1 |
| Rivers State | 2 | 4 | 2 | 2 | 1 | 2 |
| Ekiti | 0 | 0 | 0 | 0 | 0 | 0 |
| Lagos | 0 | 1 | 0 | 1 | 0 | 3 |
| Ogun | 0 | 0 | 0 | 0 | 0 | 1 |
| Ondo | 0 | 0 | 0 | 0 | 0 | 0 |
| Osun | 0 | 0 | 0 | 0 | 0 | 0 |
| Oyo | 0 | 0 | 0 | 1 | 0 | 2 |

0

2

4

6

8

10

12

14

Benue

FCT Abuja

Kogi

Kwara

Nasarawa

Niger State

Plateau

Adamawa

Bauchi

Borno

Gombe

Taraba

Yobe

Jigawa

Kaduna

Kano

Katsina

Kebbi

Sokoto

Zamfara

Abia

Anambra

Ebonyi

Enugu

Imo

Akwa ibom

Bayelsa

Cross River

Delta

Edo

Rivers State

Ekiti

Lagos

Ogun

Ondo

Osun

Oyo

**Flood Drivers Specifics by State**

Building on Natural Waterways

Climate Change

Deforestation and Land Degradation

Heavy Rainfall

Poor Infrastructure

River Overflow - Dam Release

## *Table E: Results of Flood Risk Qualitative Study: Relevance to Logistics and Health Outcomes by Zone (Source: Authors own work)*

|  | Access to Essentials | Emergency Relief Distribution | Recovery Planning | Warehousing | Transportation or  Infrastructure Impact |
| --- | --- | --- | --- | --- | --- |
| North-Central | 6 | 4 | 5 | 2 | 5 |
| North-East | 5 | 2 | 5 | 1 | 3 |
| North-West | 6 | 3 | 7 | 2 | 6 |
| South-East | 2 | 1 | 2 | 0 | 1 |
| South-South | 4 | 2 | 4 | 1 | 4 |
| South-West | 4 | 4 | 3 | 0 | 2 |

## *Table/Figure F1, F2: Results of Flood Risk Qualitative Study: Relevance to Logistics and Health Outcomes by State (Source: Authors own work)*

|  | Access to Essentials | Emergency Relief Distribution | Recovery Planning | Transportation or Infrastructure Impact | Warehousing |
| --- | --- | --- | --- | --- | --- |
| Benue | 2 | 1 | 0 | 1 | 0 |
| FCT Abuja | 1 | 1 | 0 | 1 | 0 |
| Kogi | 1 | 1 | 0 | 1 | 0 |
| Kwara | 0 | 0 | 0 | 0 | 0 |
| Nasarawa | 2 | 1 | 0 | 1 | 0 |
| Niger State | 2 | 1 | 1 | 1 | 0 |
| Plateau | 1 | 1 | 0 | 1 | 0 |
| Adamawa | 1 | 0 | 0 | 0 | 0 |
| Bauchi | 1 | 0 | 0 | 0 | 0 |
| Borno | 0 | 0 | 0 | 0 | 0 |
| Gombe | 0 | 0 | 1 | 0 | 0 |
| Taraba | 1 | 0 | 0 | 0 | 0 |
| Yobe | 0 | 0 | 0 | 0 | 0 |
| Jigawa | 2 | 2 | 1 | 2 | 0 |
| Kaduna | 2 | 1 | 0 | 2 | 0 |
| Kano | 3 | 1 | 1 | 1 | 0 |
| Katsina | 2 | 1 | 1 | 1 | 0 |
| Kebbi | 1 | 1 | 0 | 1 | 0 |
| Sokoto | 2 | 1 | 0 | 2 | 0 |
| Zamfara | 1 | 1 | 0 | 1 | 0 |
| Abia | 1 | 1 | 0 | 1 | 0 |
| Anambra | 1 | 0 | 0 | 0 | 0 |
| Ebonyi | 1 | 1 | 0 | 1 | 0 |
| Enugu | 0 | 0 | 1 | 0 | 0 |
| Imo | 0 | 0 | 0 | 0 | 0 |
| Akwa ibom | 1 | 1 | 0 | 1 | 0 |
| Bayelsa | 2 | 1 | 1 | 1 | 0 |
| Cross River | 0 | 0 | 0 | 0 | 0 |
| Delta | 1 | 1 | 0 | 1 | 0 |
| Edo | 2 | 1 | 0 | 1 | 0 |
| Rivers State | 2 | 1 | 2 | 2 | 0 |
| Ekiti | 0 | 0 | 0 | 0 | 0 |
| Lagos | 1 | 1 | 0 | 1 | 0 |
| Ogun | 0 | 0 | 0 | 0 | 0 |
| Ondo | 0 | 0 | 0 | 0 | 0 |
| Osun | 0 | 0 | 0 | 0 | 0 |
| Oyo | 1 | 1 | 0 | 1 | 0 |

0

1

2

3

4

5

6

7

8

Benue

FCT Abuja

Kogi

Kwara

Nasarawa

Niger State

Plateau

Adamawa

Bauchi

Borno

Gombe

Taraba

Yobe

Jigawa

Kaduna

Kano

Katsina

Kebbi

Sokoto

Zamfara

Abia

Anambra

Ebonyi

Enugu

Imo

Akwa ibom

Bayelsa

Cross River

Delta

Edo

Rivers State

Ekiti

Lagos

Ogun

Ondo

Osun

Oyo

**Logistics and Health Outcomes Specifics**

**by State**

Access to Essentials

Emergency Relief Distribution

Recovery Planning

Transportation or Infrastructure Impact

Warehousing

## *Figures G1, G2: Results of Flood Risk Qualitative Study – Most Relevantly Flood-Exposed Nigerian States*

0

2

4

6

8

10

12

14

16

Kogi

Anambra

Bayelsa

Jigawa

Kano

Niger State

Adamawa

Rivers State

Benue

Gombe

Lagos

Sokoto

Taraba

Yobe

Delta

Kaduna

Zamfara

Edo

FCT Abuja

Katsina

Nasarawa

Ogun

Oyo

Abia

Akwa ibom

Bauchi

Ebonyi

Enugu

Imo

Kebbi

Ondo

Osun

Plateau

Borno

Cross River

Ekiti

Kwara

**Most Relevantly Flood**

**-**

**Exposed Nigerian States**

© Microsoft, OpenStreetMap

Powered by Bing

4

2

14

0

2

6

1

5

1

0

4

4

4

8

3

8

2

1

4

3

1

9

1

1

1

1

9

0

3

2

5

0

2

1

1

2

**Most Relevantly Flood**

**-**

**Exposed Nigerian States**


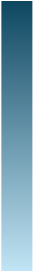


0

14

Reference

## *Table H: Expert Survey Themes Operationalisation (Source: Authors own work)*

| Theme | Definition | Observable Action | Survey Questions |
| --- | --- | --- | --- |
| Feasibility | Feasibility refers to the capability of finding solutions that are suitable for the conditions and appropriate for intended use [46] | Are the existing warehouses usable for medical prepositioning? | 1. What is your primary area of work or expertise related to flood preparedness or emergency logistics in Nigeria? 2. NEMA reports 16 warehouses for emergency prepositioning across 14 locations. Have you ever worked with or been in contact with one or more of these warehouses during a flood response or preparedness operation? 3. Have you ever worked on or observed a flood response where a NEMA warehouse played a key logistical role? 4. To your knowledge, are the current NEMA warehouses suitable for storing medical consumable supplies (e.g., emergency kits, essential drugs, vaccines)? 5. Which infrastructure limitations have you encountered or are aware of in the current warehouse network? (Select all that apply) 6. In your experience or opinion, which of the current warehouse locations are most operationally ready to serve as medical supply hubs? |
| Effectiveness | Effectiveness refers to the extent to which a specific action/object, when used under ordinary or routine circumstances, does what it is intended to do [47] | Which warehouse locations are most useful in real operations? | 1. Based on your experience or knowledge, which NEMA warehouse locations have been the most effective in supporting flood-related emergency operations? 2. Which of the following factors have made certain warehouse locations more effective during flood responses? (Select all that apply) 3. In your experience, have any warehouse locations been consistently delayed, inaccessible, or underutilised during real flood emergencies? 4. How would you rate the overall effectiveness of the NEMA warehouse network in supporting medical supply distribution during flood emergencies? 5. In your opinion, what are the key improvements needed to increase the effectiveness of warehouse operations during floods? (Prompt: Think about staffing, equipment, funds, coordination, location, or any other factor.) |
| Coverage | Coverage refers to the extent to which logistics networks reach the largest number of people, avoid shortages, theft, and waste, and provide equitable access to aid [48] | Are there geographic or population gaps that current warehouses don’t cover? | 1. In your opinion, are there regions or states in Nigeria that are frequently affected by flooding but lack timely access to medical supplies from existing warehouses? 2. Which of the following zones do you believe are underserved by the current NEMA warehouse network during flood emergencies? 3. Which of the following zones do you believe has warehousing capacity but remains underserved by the current NEMA warehouse network during flood emergencies? 4. Have you ever witnessed or heard of a flood response operation that was delayed due to lack of proximity to a warehouse? 5. In your professional view, does the current warehouse network provide adequate national coverage for prepositioning medical supplies during floods? 6. Are there specific high-risk flood zones that you believe should be prioritised for future warehouse placement? |
| Optimisation | Optimisation in logistics refers to the extent to which improving processes through various means to enhance efficiency [49] | Based on practical experience, how many locations can be sustained and are actually needed? | 1. In your professional opinion, what is the minimum number of warehouse locations needed per zone to ensure effective flood response for medical supplies across Nigeria? 2. Do you believe that the current number of 16 NEMA warehouses is: 3. If resources were severely limited and only a very reduced number of warehouses could be maintained, what would be the realistic minimum number of locations for sustainable and effective operation? 4. From your experience, is it more effective to have: 5. In your understanding, how is the NEMA warehouse network currently structured in terms of logistics coordination? 6. Do you believe there is effective coordination between national, state, and local authorities in managing warehouse logistics during flood responses? 7. To what extent are the current warehouses integrated into the national health supply chain system (e.g., cold chain, last-mile delivery, medical inventory systems)? |
| Prioritisation | Prioritisation refers to the process of ranking or selecting options based on strategic importance and constraints [50] | If we had to pick 10, which should be kept? | 1. If only 10 NEMA warehouse locations could be maintained for flood-related medical supply prepositioning, which locations would you prioritise? Rank 1 to 14 2. What key criteria did you use to select those 10 warehouse locations? (Select all that apply) 3. Which existing warehouse locations, in your view, are least essential and could be de-prioritised if necessary? (Select all that apply) 4. Should warehouse prioritisation be based more on: 5. Do you believe there is a need to adjust warehouse locations regularly based on changing flood patterns and population movement? |

## *Table I: Respondents Data (Source: Authors own work)*

| Total Respondents | 59 |
| --- | --- |
| Consent to do the survey | 58 |
| Total Completed Surveys | 34 |
| **Field of work** | |
| Disaster response coordination | 5 |
| Humanitarian logistics or medical supply chain | 1 |
| Public health planning or emergency health services | 16 |
| GIS analysis or flood risk mapping | 1 |
| Infrastructure or urban planning | 0 |
| Academic research on flooding or disaster management | 4 |
| Government policymaking or agency work (e.g., NEMA, NIHSA) | 4 |
| NGO or international organisation programme management | 1 |
| Private sector logistics or warehousing | 0 |
| Other (please specify) | 2 |
| Prefer Not to Say | 6 |
| Desease Survailance | 1 |
| **Direct Experience with NEMA Warehouses** | |
| Abuja | 0 |
| Maiduguri | 3 |
| Jos | 1 |
| Lagos | 1 |
| Kaduna | 0 |
| Enugu | 0 |
| Owerri | 1 |
| Kano | 1 |
| Gombe | 0 |
| Ekiti | 0 |
| Port Harcourt | 1 |
| Sokoto | 0 |
| Yola | 1 |
| Minna | 1 |
| I have never worked with any NEMA warehouse | 24 |
| Other (please specify) | 0 |
| Prefer Not to Say | 1 |
| **Number of respondents who had direct work experience with NEMA Warehouses** | |
| Worked in a warehouse | 5 |
| Been in contact with a warehouse but not worked | 1 |

## *Table J: Results of Survey Study and Qualitative Study – Geographic Focus Data Report (Source: Authors own work)*

[ ≥5=High
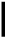
 2−4=Moderate
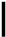
 1=Low
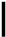
 0=Negligible]

|  |  |  |  |  |  |  |  |  |  |  |  |  |  |  |  |  |  |  |  |  |  |  |  |  |  |  |  |  |  |  |  |  |  |  |  |  |  |
| --- | --- | --- | --- | --- | --- | --- | --- | --- | --- | --- | --- | --- | --- | --- | --- | --- | --- | --- | --- | --- | --- | --- | --- | --- | --- | --- | --- | --- | --- | --- | --- | --- | --- | --- | --- | --- | --- |
| State | FCT Abuja | Nasarawa | Kogi | Yobe | Ogun | Oyo | Lagos | Edo | Borno | Niger State | Anambra | Jigawa | Kaduna | Bayelsa | Kano | Gombe | Sokoto | Taraba | Zamfara | Katsina | Kwara | Adamawa | Rivers State | Benue | Abia | Bauchi | Ebonyi | Enugu | Imo | Kebbi | Ondo | Osun | Plateau | Delta | Ekiti | Akwa Ibom | Cross River |
| LGA | Abuja | Abuja | Abuja | Maiduguri | Lagos | Lagos | Lagos | Lagos | Maiduguri | Minna | Enugu | Kano | Kaduna | Port Harcourt | Kano | Gombe | Sokoto | Yola | Sokoto | Kano | Minna | Yola | Port Harcourt | Jos | Owerri | Gombe | Enugu | Enugu | Owerri | Sokoto | Ekiti | Ekiti | Jos | Port Harcourt | Ekiti | Port Harcourt | Port Harcourt |
| City | Abuja | Abuja | Abuja | Maiduguri | Lagos | Lagos | Lagos | Lagos | Maiduguri | Minna | Enugu | Kano | Kaduna | Port Harcourt | Kano | Gombe | Sokoto | Yola | Sokoto | Kano | Minna | Yola | Port Harcourt | Jos | Owerri | Gombe | Enugu | Enugu | Owerri | Sokoto | Ekiti | Ekiti | Jos | Port Harcourt | Ekiti | Port Harcourt | Port Harcourt |
| FloodDiv_N | 2 | 2 | 14 | 4 | 2 | 2 | 4 | 2 | 0 | 6 | 9 | 8 | 3 | 9 | 8 | 4 | 4 | 4 | 3 | 2 | 0 | 5 | 5 | 4 | 1 | 1 | 1 | 1 | 1 | 1 | 1 | 1 | 1 | 3 | 0 | 1 | 0 |
| FloodDiv_Grade | Moderate | Moderate | Very High | Moderate | Moderate | Moderate | Moderate | Moderate | Negligible | High | Very High | Very High | Moderate | Very High | Very High | Moderate | Moderate | Moderate | Moderate | Moderate | Negligible | High | High | Moderate | Low | Low | Low | Low | Low | Low | Low | Low | Low | Moderate | Negligible | Low | Negligible |
| Q4_N | 6 | 6 | 6 | 1 | 2 | 2 | 2 | 2 | 1 | 0 | 0 | 0 | 0 | 0 | 0 | 0 | 0 | 0 | 0 | 0 | 0 | 0 | 0 | 0 | 0 | 0 | 0 | 0 | 0 | 0 | 0 | 0 | 0 | 0 | 0 | 0 | 0 |
| Q4_Grade | High | High | High | Low | Moderate | Moderate | Moderate | Moderate | Low | Negligible | Negligible | Negligible | Negligible | Negligible | Negligible | Negligible | Negligible | Negligible | Negligible | Negligible | Negligible | Negligible | Negligible | Negligible | Negligible | Negligible | Negligible | Negligible | Negligible | Negligible | Negligible | Negligible | Negligible | Negligible | Negligible | Negligible | Negligible |
| Q5_N | 2 | 2 | 2 | 2 | 2 | 2 | 2 | 2 | 2 | 1 | 0 | 0 | 1 | 0 | 0 | 0 | 0 | 0 | 0 | 0 | 1 | 0 | 0 | 0 | 0 | 0 | 0 | 0 | 0 | 0 | 0 | 0 | 0 | 0 | 0 | 0 | 0 |
| Q5_Grade | Moderate | Moderate | Moderate | Moderate | Moderate | Moderate | Moderate | Moderate | Moderate | Low | Negligible | Negligible | Low | Negligible | Negligible | Negligible | Negligible | Negligible | Negligible | Negligible | Low | Negligible | Negligible | Negligible | Negligible | Negligible | Negligible | Negligible | Negligible | Negligible | Negligible | Negligible | Negligible | Negligible | Negligible | Negligible | Negligible |
| Q21_N | 1 | 1 | 1 | 2 | 1 | 1 | 1 | 1 | 2 | 2 | 0 | 0 | 0 | 0 | 0 | 0 | 0 | 0 | 0 | 0 | 2 | 0 | 0 | 0 | 0 | 0 | 0 | 0 | 0 | 0 | 0 | 0 | 0 | 0 | 0 | 0 | 0 |
| Q21_Grade | Low | Low | Low | Moderate | Low | Low | Low | Low | Moderate | Moderate | Negligible | Negligible | Negligible | Negligible | Negligible | Negligible | Negligible | Negligible | Negligible | Negligible | Moderate | Negligible | Negligible | Negligible | Negligible | Negligible | Negligible | Negligible | Negligible | Negligible | Negligible | Negligible | Negligible | Negligible | Negligible | Negligible | Negligible |
| Q11_N | 8 | 8 | 8 | 3 | 0 | 0 | 0 | 8 | 3 | 8 | 1 | 1 | 1 | 8 | 1 | 3 | 1 | 3 | 1 | 1 | 8 | 3 | 8 | 8 | 1 | 3 | 1 | 1 | 1 | 1 | 0 | 0 | 8 | 8 | 0 | 8 | 8 |
| Q11_Grade | High | High | High | Moderate | Negligible | Negligible | Negligible | High | Moderate | High | Low | Low | Low | High | Low | Moderate | Low | Moderate | Low | Low | High | Moderate | High | High | Low | Moderate | Low | Low | Low | Low | Negligible | Negligible | High | High | Negligible | High | High |
| Q10_N | 0 | 0 | 4 | 0 | 0 | 0 | 2 | 1 | 0 | 4 | 1 | 1 | 0 | 1 | 1 | 0 | 0 | 0 | 0 | 0 | 2 | 2 | 1 | 1 | 0 | 0 | 0 | 0 | 0 | 0 | 0 | 0 | 0 | 1 | 0 | 1 | 2 |
| Q10_Grade | Negligible | Negligible | Moderate | Negligible | Negligible | Negligible | Moderate | Low | Negligible | Moderate | Low | Low | Negligible | Low | Low | Negligible | Negligible | Negligible | Negligible | Negligible | Moderate | Moderate | Low | Low | Negligible | Negligible | Negligible | Negligible | Negligible | Negligible | Negligible | Negligible | Negligible | Low | Negligible | Low | Moderate |
| Q14_N | 8 | 8 | 8 | 3 | 0 | 0 | 0 | 7 | 3 | 8 | 1 | 1 | 1 | 7 | 1 | 3 | 1 | 3 | 1 | 1 | 8 | 3 | 7 | 8 | 1 | 3 | 1 | 1 | 1 | 1 | 0 | 0 | 8 | 7 | 0 | 7 | 7 |
| Q14_Grade | High | High | High | Moderate | Negligible | Negligible | Negligible | High | Moderate | High | Low | Low | Low | High | Low | Moderate | Low | Moderate | Low | Low | High | Moderate | High | High | Low | Moderate | Low | Low | Low | Low | Negligible | Negligible | High | High | Negligible | High | High |
| Comp_N | 12 | 12 | 10 | 9 | 8 | 8 | 6 | 6 | 6 | 4 | 3 | 3 | 3 | 2 | 2 | 2 | 2 | 2 | 2 | 2 | 2 | 1 | 1 | 1 | 1 | 1 | 1 | 1 | 1 | 1 | 1 | 1 | 1 | 0 | 0 | -1 | -3 |
| Comp_Grade | High | High | High | High | High | High | High | High | High | Moderate | Moderate | Moderate | Moderate | Moderate | Moderate | Moderate | Moderate | Moderate | Moderate | Moderate | Moderate | Low | Low | Low | Low | Low | Low | Low | Low | Low | Low | Low | Low | Negligible | Negligible | Negligible | Negligible |
